# Analysis of BRAF and NRAS Mutation Status in Advanced Melanoma Patients Treated with Anti-CTLA-4 Antibodies: Association with Overall Survival?

**DOI:** 10.1371/journal.pone.0139438

**Published:** 2015-10-01

**Authors:** Joanna Mangana, Phil F. Cheng, Katja Schindler, Benjamin Weide, Ulrike Held, Anna L. Frauchiger, Emanuella Romano, Katharina C. Kähler, Sima Rozati, Markus Rechsteiner, Holger Moch, Olivier Michielin, Claus Garbe, Axel Hauschild, Christoph Hoeller, Reinhard Dummer, Simone M. Goldinger

**Affiliations:** 1 Department of Dermatology, University Hospital Zurich, Zurich, Switzerland; 2 Department of Dermatology, Medical University of Vienna, Vienna, Austria; 3 Department of Dermatology, University Hospital Tübingen, Tübingen, Germany; 4 Institute of Surgical Pathology, University Hospital Zurich, Zurich, Switzerland; 5 Department of Oncology CHUV Lausanne, Lausanne, Switzerland; 6 Department of Dermatology, University Hospital Schleswig-Holstein Campus Kiel, Kiel, Germany; 7 Department of Dermatology, Stanford University School of Medicine, Stanford, United States of America; 8 Horten Centre for Patient Oriented Research and Knowledge Transfer, University Hospital Zurich, Zurich, Switzerland; University of California Davis, UNITED STATES

## Abstract

Ipilimumab and tremelimumab are human monoclonal antibodies (Abs) against cytotoxic T-lymphocyte antigen-4 (CTLA-4). Ipilimumab was the first agent to show a statistically significant benefit in overall survival in advanced melanoma patients. Currently, there is no proven association between the BRAFV600 mutation and the disease control rate in response to ipilimumab. This analysis was carried out to assess if BRAFV600 and NRAS mutation status affects the clinical outcome of anti-CTLA-4-treated melanoma patients. This is a retrospective multi-center analysis of 101 patients, with confirmed BRAF and NRAS mutation status, treated with anti-CTLA-4 antibodies from December 2006 until August 2012. The median overall survival, defined from the treatment start date with the anti-CTLA-4. Abs-treatment to death or till last follow up, of BRAFV600 or NRAS mutant patients (n = 62) was 10.12 months (95% CI 6.78–13.2) compared to 8.26 months (95% CI 6.02–19.9) in BRAFV600/NRASwt subpopulation (n = 39) (p = 0.67). The median OS of NRAS mutated patients (n = 24) was 12.1 months and although was prolonged compared to the median OS of BRAF mutated patients (n = 38, mOS = 8.03 months) or BRAFV600/NRASwt patients (n = 39, mOS = 8.26 months) the difference didn’t reach statistical significance (p = 0.56). 69 patients were able to complete 4 cycles of anti-CTLA-4 treatment. Of the 24 patients treated with selected BRAF- or MEK-inhibitors, 16 patients received anti-CTLA 4 Abs following either a BRAF or MEK inhibitor with only 8 of them being able to finish 4 cycles of treatment. Based on our results, there is no difference in the median OS in patients treated with anti-CTLA-4 Abs implying that the BRAF/NRAS mutation status alone is not sufficient to predict the outcome of patients treated with anti-CTLA-4 Abs.

## Introduction

Melanoma has been long considered an immunogenic cancer based on reports of spontaneous regression and some tumor responses after immune-stimulating agent treatment [1–3]. Taking this into consideration, multiple efforts in cytokine therapy, tumor vaccines, and adoptive immunotherapy have been pursued to harness the immune response to tackle melanoma but have had slow progress over the decades [1]. These attempts were limited due to the innate mechanisms of the immune system preventing its over-activation against self-antigens and as well to some serious toxic side effects. Inorder to turn on the immune system against cancer another promishing approach, focused on blocking the negative-regulator of T-cell responses, the cytotoxic T-lymphocyte-associate antigen (CTLA-4), which marked a new era in the treatment of advanced melanoma and oncoimmunotherapy [4].

Both ipilimumab and tremelimumab are fully human monoclonal antibodies (Abs) against CTLA-4. Ipilimumab was the first agent to show a statistically significant benefit in overall survival (OS) in stage IV melanoma patients both in first and second line settings [5,6]. Although long-durable responses have been reported in a subpopulation of patients, the response rates are commonly low and currently there are no molecular markers to predict for responders. On the other hand, tremelimumab failed to significantly improve OS over standard chemotherapy [7]. This was partly explained due to patients’ selection criteria, as patients with LDH levels greater than 2x upper limit of normal (2xULN) were excluded according to the study protocol. Another explanation was the unintended crossover to ipilimumab in the control arm, as crossover to tremelimumab was not allowed within the study protocol.

The BRAF oncogene is mutated in approximately 50% of metastatic melanomas [8–11]. Over 90% of the mutations result in substitution of the valine in position 600 (thus V600), which allows for constitutive activation of the RAS-RAF-MEK-MAPK pathway [12]. This finding made the RAS-RAF-MEK-MAPK pathway the most promising target in melanoma research and led to the development of targeted therapy against mutated BRAFV600 which resulted in a treatment-breakthrough with impressive clinical responses and significant prolongation of progression-free-survival (PFS) and OS in the majority of advanced melanoma patients in clinical trials [13–16]. In addition to BRAF, NRAS is mutated in 15–25% of all melanomas, most frequently in exon 1 (G12) and exon 2 (Q61) [17]. These activating NRAS mutations result also in a constitutive activation of the MAP-kinase signal transduction pathway (MAPK pathway) [18]. It is of interest to note that somatic mutations in the BRAF and NRAS gene are mutually exclusive [19], thus constitutive activation of the MAPK pathway occurs in approximately 65–75% of all melanoma tumors.

There is evidence that upon activation of the MAP-kinase pathway an immunosuppressive phenotype of the tumor is promoted [20]. Whether the mutation status directly correlates with the clinical outcome remains controversial. Long et al. showed that the presence of an activated mutation in the BRAF oncogene was associated with a worse clinical outcome but no impact at the disease free interval [21]. In a small cohort of advanced melanoma patients treated with bevacizumab and temozolomide, both response and OS were proved to be significantly higher in the wild-type (wt) population [22]. Nevertheless, and in the retrospective setting, no trend for a shorter survival in BRAF-mutant patients could be determined [8,23–29]. Recently, the presence of an NRAS mutation was identified as an independent factor for a worse outcome in metastatic melanoma [18]. On the other hand Davies et al. suggested NRAS status as a possible biomarker for response to high dose (HD) interleukin-2 treatment (IL2) [30].

Anti-CTLA-4 Abs are likely to be equally efficacious in both BRAF mutated (BRAFmut) and wt patients, as they act independently of the MAPK signaling pathway. To date, no association between the BRAFV600E mutation and the disease control rate (DCR) after treatment with ipilimumab was determined [31]. However, according to preliminary unpublished results treatment with ipilimumab or the anti-PD1-Ab nivolumab is associated with superior clinical benefit (increased response rate) in patients harboring the NRAS mutation with no impact in the PFS or OS [32].

To our knowledge this is the first study to assess if both BRAFV600 and NRAS mutation status affect the clinical outcome of stage IV melanoma patients treated with anti-CTLA-4 antibodies. We sought to test this hypothesis in a retrospective accrued multicenter cohort of advanced melanoma patients.

## Materials and Methods

### Patient Selection and Data Collection

Patients with stage IV metastatic melanoma (American Joint Committee on Cancer, AJCC) having been treated with anti-CTLA-4 Abs in the period between December 2006 and October 2012 from 5 international melanoma centers with evaluated BRAF and NRAS mutation status formed the study cohort. Patients with non-resectable stage IIIC AJCC were not included.

Geographic and histopathologic data including gender, age, melanoma type, localization, Breslow’s depth, mitotic rate, and presence or absence of ulceration were assessed. Data on treatment after diagnosis of metastatic disease, including development of new sites of distant metastases, surgery, systemic therapies, radiotherapy, and survival status were retrospectively collected for all patients.

Written informed consent for tissue storage including retrospective analysis with collection of Clinical/laboratory/histology information was previously approved by local ethics committee (Kantonale Ethikkomission Zürich Biobank/Sammlung von Tumorgewebe, KEK-ZH-Nr. 647). The clinical information was anonymized and de-identified prior to analysis.

Data were classified with dichotomous variables (yes or no) or coded with the quantity of treatments and metastatic sites. All patients had to be treated at least with one infusion with an anti-CTLA-4 antibody for a maximum of four infusions (ipilimumab 10mg/kg or 3mg/kg or tremelimumab 15mg/kg).

OS was defined as the length of time in months from the start of treatment to either death or last follow up (analysis accounted for censored survival times). Median OS (mOS) in stage IV disease was defined as the length of time in months from the detection of the first distant metastasis to death or till last follow up. Treatment duration (TD) was defined as the interval between the initiation of treatment and treatment discontinuation due to either disease progression or toxicity and was determined in months (rounded up to a decimal).

### Anti-CTLA-4 Antibodies

Eligible patients included BRAFV600- or NRAS-mutated and BRAFV600 and NRAS wild-type that were treated with the antibody ipilimumab (formerly MDX-010 and MDX-101, registered as Yervoy; Bristol Myers Squibb, NY, USA) 10mg/kg every three weeks within the registry trial MDX-020 (clinicaltrials.gov NCT00094653) and 3mg/kg every three weeks within the available compassionate use program and after the registration in Europe and Switzerland. Re-induction was allowed in those patients with progression after disease stabilization. Moreover, four patients were treated with tremelimumab (formerly ticilimumab, CP-675,206; Pfizer, NY, USA) 15 mg/kg every 12 weeks for a maximum of four infusions.

### Cut-off values LDH and S100B

LDH and S100B were assessed from the patients’ serum. Normal LDH and S100B levels were defined as lower or equal to the reference cut-off of 480 UI/l and 0.2 ug/l respectively, as defined by the normal ranges of the biochemical and immunological laboratories of the University Hospital of Zurich.

### Mutation Status Assessment

Sections from archival paraffin-embedded samples were tested for BRAF (exon 15) and NRAS status (exon 2 and 3) at the local departments of dermatology and pathology. The study cohort included patients treated in clinical trials registered at clinicaltrials.gov (NCT Numbers of the trials NCT00094653 and NCT00257205). These patients were tested at central labs with more extensive sequencing. All BRAFwt melanomas were additionally tested for NRAS status. In the situation where the BRAF and NRAS status was unknown, the mutation status analysis was performed according to standard procedures at the local laboratories of each center.

### Statistical Analysis

Descriptive statistics are presented as percentages of total for categorical variables and as median for continuous and ordinal variables. For survival time, summary measures include median overall survival and 95% confidence interval. Chi-squared test and Wilcoxon rank sum test were used for group comparisons for categorical and continuous parameters. The log rank test was used for the comparison of survival curves. A Cox proportional hazards model was used to estimate the adjusted hazard ratio (HR) for treatment duration (TD). Potential confounders included the variables mutation status and BRAF / MEK inhibitors. All p-values were for two-sided test and p<0.05 was considered statistical significance. The analysis was carried out with Graphpad Prism5 and R-project [33].

## Results

A total of one hundred and one (n = 101) AJCC Stage IV melanoma patients treated with anti-CTLA-4 Abs formed the study cohort. Either loco-regional or distant metastases were used for BRAF and NRAS mutation testing. One patient had a pW616R-BRAF mutation and a pG10E mutation in the NRAS oncogene and was classified as BRAFV600/NRASwt. Thirty eight patients (38%) were mutant for BRAF and twenty four (24%) for NRAS for a total of sixty two patients with a BRAF or NRAS mutation. Thirty-nine patients (39%) lacked BRAF or NRAS mutation and were termed as BRAFV600/NRASwt. As of January 2014, eighty three patients (82%) had passed away and of the eighteen (18%) still alive four were lost to follow up.

### Anti-CTLA-4 Antibodies and Treatment Characteristics

**Fig 1:**
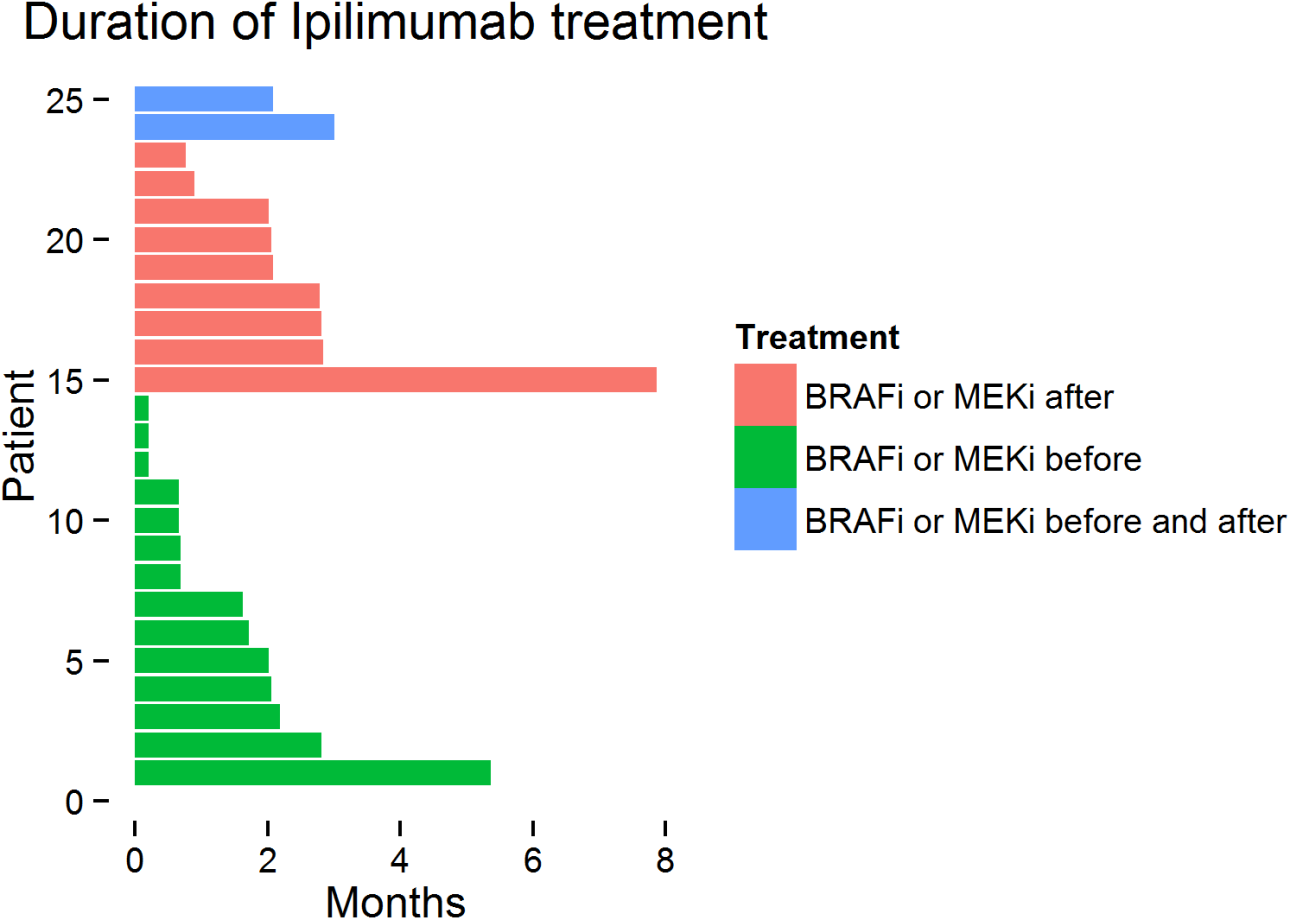
Overview of all anti-CTLA-4 patients according to treatment duration and treatment with BRAF or MEK inhibitors either prior or after anti-CTLA-4Abs. Each bar represents one patient.

Fifteen patients (15%) received anti-CTLA-4 Abs as first line treatment. The median TD in the complete study cohort (n = 101) was 2.38 months. Sixty-nine patients (69%) were able to complete 4 cycles of anti-CTLA-4 treatment. After completion of anti-CTLA-4 treatment, nine patients were subsequently treated with BRAF or MEK inhibitors. Sixteen patients received either a BRAF or MEK inhibitor prior to anti-CTLA-4 treatment with only eight of them (50%) being able to finish 4 cycles of treatment; two of the patients were re-exposed to BRAF or MEK inhibition treatment upon progression to anti-CTLA-4 Abs. The rest of the patients (n = 8) discontinued the treatment either due to progression (n = 7) or due to adverse events (n = 1) (Fig 1).

### Mutation Status and Patients’ Characteristics

Histopathologic information such as melanoma subtype, localization of primary tumor, and tumor thickness were known for all patients, while the presence or absence of ulceration was available in 34% of the patients (n = 34). Patients’ characteristics, demographics and features of primary and metastatic melanoma in regard to mutation status are listed in Table 1.

**Table 1:** Patient demographics and primary melanoma characteristics.

Characteristics	BRAFV600 or NRAS mut N = 62	BRAF/NRAS wild type N = 39
**Age**		
Median	54.7	60.1
**Gender**		
Male	29 (47%)	23 (59%)
Female	33 (53%)	16 (51%)
**Histopathologic subtype**		
Superficial spreading	11 (17.8%)	3 (7.7%)
Nodular	24 (38.8%)	5 (12.8%)
Acral lentiginous	3 (4.8%)	8 (20.5%)
Lentigo maligna	0 (0%)	1 (2.6%)
Desmoplastic	0 (0%)	1 (2.6%)
Amelanotic	1 (1.6%)	1 (2.6%)
Mucosal	2 (3.2%)	3 (7.7%)
Uveal	0 (0%)	2 (5.1%)
Other*	5 (8%)	0 (0%)
Unknown	16 (25.8%)	15 (38.4%)
**Breslow (mm)**		
0.01–1.0	9 (14.5%)	6 (15.4%)
1.01–2	10 (16.1%)	5 (12.8%)
2.01–4	14 (22.6%)	3 (7.7%)
>4	14 (22.6%)	14 (35.9%)
Unknown	15 (24.2%)	11 (28.2%)
**Stage at first diagnosis**		
0	0 (0%)	0 (0%)
I	8 (12.9%)	6 (15.4%)
II	19 (30.6%)	12 (30.8%)
III	21 (33.9%)	13 (33.3%)
IV	6 (9.7%)	3 (7.7%)
Unknown	8 (12.9%)	5 (12.8%)

* including polypoid, solid and melanoma ex naevo.

BRAFV600 or NRAS mutated patients were younger at time of first diagnosis (median age = 53, p = 0.004) than the BRAFV600/NRASwt patients (median age 63). No difference in sex distribution among the two sub-groups was found (p = 0.322). Analyzing the site of the primary antecedent melanoma, we found that BRAFV600 or NRASmut melanomas were more frequently located at the trunk and less frequently at the acra, compared to BRAFV600/NRASwt (p = 0.016) (Table 2).

**Table 2:** Association of mutation status with age and features of metastatic and antecedent primary melanoma.

Demographic/disease characteristics	BRAFV600 or NRAS mut N = 62 (61%)	BRAF/NRAS wt N = 39 (39%)	p-value
**Age**			
Median	53 (42–62)	63 (55–70)	0.004*
**Gender**			
Male	29 (47%)	23 (59%)	
**Localization**			
Acra	3 (5%)	8 (21%)	0.016**
Extremities	16 (26%)	5 (13%)	
Trunk	15 (24%)	5 (15%)	
**LDH elevated**	8 (16%), missing 13	8 (26%), missing 8	0.456**
**S100 elevated**	23 (58%), missing 22	10 (4%), missing 12	0.163**

* Wilcoxon test

** Chi-squared test.

At stage IV disease forty two patients were staged as M1a or M1b at baseline. The clinical characteristics of stage IV disease are listed in Table 3. Forty-one patients (41%) developed brain metastases at some point of stage IV disease. The LDH and S100B levels at stage IV disease were known in sixty one patients. BRAFV600 or NRASmut patients had higher levels of S100 and lower levels of LDH, but the difference was not statistically significant (p = 0.26, p = 0.45, respectively).

**Table 3:** Clinical characteristics at stage IV disease according to mutation status.

Characteristics	BRAFV600 or NRAS mut N = 62 (61%)	BRAF/NRAS wild-type N = 39 (39%)
**LDH**		
Normal	30	19
Elevated	8	8
Unknown	24	12
**S100**		
Normal	17	17
Elevated	23	10
Unknown	22	12
**Metastasis category**		
M1a	10	8
M1b	13	11
M1c	38	17
Unknown	1	3
**CNS involvement**		
Yes	27	14
No	35	25

### Mutation Status and Clinical Outcome

**Fig 2:**
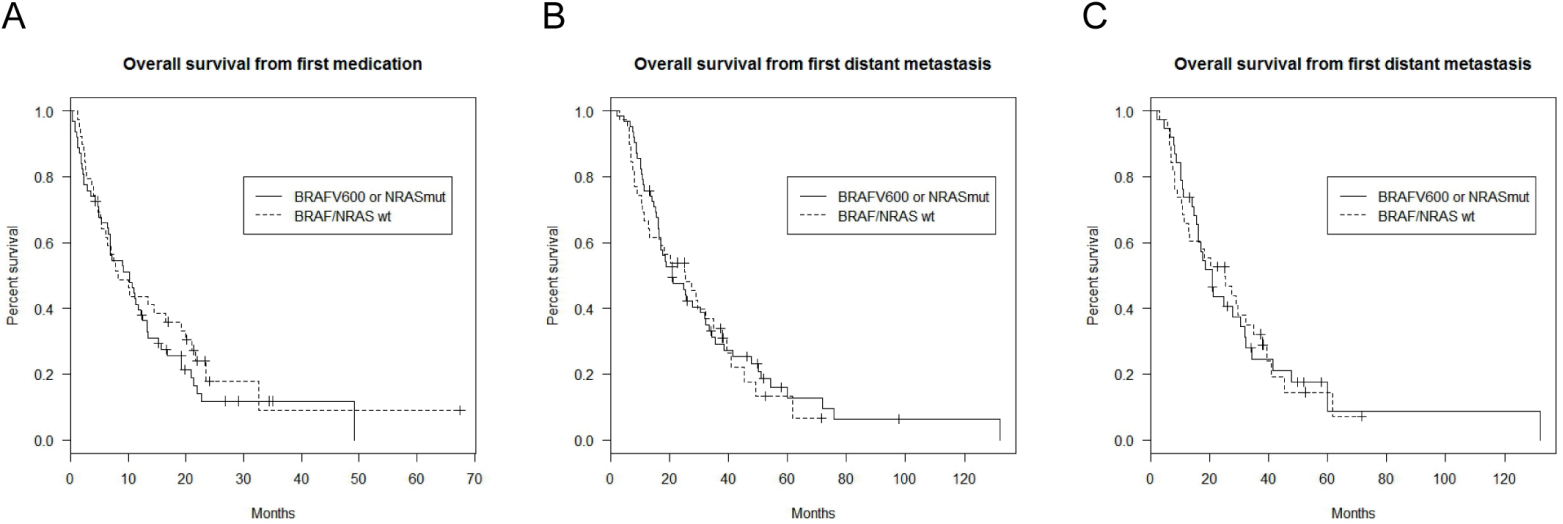
Impact of mutation status on overall survival (OS), defined from initiation of anti-CTLA-4 treatment (Fig 2a) and from stage IV melanoma (Fig 2b) according to BRAF and NRAS mutation status in all patients (n = 101). The Fig 2c represents the OS according to mutation status from diagnosis of metastatic melanoma in the subgroup of patients (n = 76) with no access to BRAF/MEK inhibition treatment.

As pointed in the material and methods section, OS was defined from the date of therapy initiation with the anti-CTLA-4 Abs treatment to death or till last follow up. In the complete study cohort (n = 101), the median OS was 10.08 months. BRAFV600 or NRASmut patients had a prolonged mOS (mOS = 10.12months, 95% CI 6.78–13.2) compared to BRAFV600/NRASwt patients (mOS = 8.26 months, 95% CI 6.02–19.9) but did not differ significantly (p = 0.67) (Fig 2A). This difference remained non-significant in a multivariate analysis adjusted for age and gender (Table 4). Also for patients with known LDH and S100 serum levels, there was no statistically significant difference in the OS when adjusting for high LDH serum levels (n = 80, HR = 1.52, 95% CI 0.8–2.7, p = 0.152) and high S100 levels (n = 67, HR = 1.570, 95% CI 0.89–2.77, p = 0.120).

**Table 4:** Multivariate Analysis of overall survival (OS) from first medication in all patients (n = 101) adjusted for age and gender.

All patients, n = 101			
Co-variate	Hazard Ratio	95% CI	p-value
Mutation	0.80	0.50–1.28	0.36
Gender (female = 0)	1.06	0.69–1.63	0.80
Age	1.00	0.98–1.02	0.82

The mOS of NRAS mutated patients (n = 24) was 12.1 months and although it was longer in absolute numbers compared to the mOS of BRAF mutated patients (n = 38, mOS = 8.03 months) or BRAFV600/NRASwt patients (n = 39, mOS = 8.26months) the difference did not reach statistical significance (p = 0.56).

MOS in Stage IV disease was 24.7 months (n = 101). The 1-year, 2- and 3-year survival of the whole study population were 72% (n = 73, 95% CI, 0.62–0.80), 47% (n = 47, 95% CI (0.37–0.57) and 27% (n = 27, 95% CI 0.18–0.36) respectively. The mOS in Stage IV of BRAFV600 or NRASmut patients (n = 62) was 20.9 months (95% CI 16.4–32) and was not statistically significant compared to BRAFV600/NRASwt patients (n = 39) (mOS = 25.4 months, p = 0.719, HR = 0.91, 95% CI 13–30.3) (Fig 2B).

Upon the introduction of selective kinase inhibitors in the melanoma market, the standard of care of melanoma patients has changed. In the BRAFV600 or NRASmut subgroup, twenty five patients (39%) received a BRAF or MEK inhibitor, and with the exception of vemurafenib, mostly within clinical trials. Reasons for not obtaining targeted therapy were limited access to clinical trials, since our study cohort included patients even from 2006. Other reasons include ineligibility (eg. poor performance status or presence of brain metastases), rapid disease progression and patient preference. Apart from a favorable prognosis after targeted therapy, an ECOG performance status less than or equal to 1 is mandated for trial eligibility, which made an unbiased comparison within the BRAFV600 or NRASmut subgroup not possible. Consequently to avoid bias, patients who received treatment with kinase inhibitors were excluded from the complete cohort of patients. In this subgroup (n = 76), mOS in Stage IV disease was 20.9 months (95% CI 15.9–32.1) in the BRAFV600 or NRASmut arm and 25.1 months (95% CI 12.8–37.7) in the BRAFV600/NRASwt arm (p = 0.96, HR = 0.987) (Fig 2C). When sub-setting for the long survivors (patients with an OS>18months, n = 51) no difference in the mOS in Stage IV was determined (p = 0.99).

In the BRAFV600 or NRASmut cohort the TD was 2.44 months and did not differ significantly compared to the TD of the BRAFV600/NRASwt cohort (TD = 2.55 months, p = 0.84). In the Cox proportional hazards regression model for OS in the whole study cohort (n = 101), and after accounting for treatment with BRAF or MEK inhibitors and mutation status, the determinant TD had a statistically significant association with overall survival in Stage IV disease (HR = 0.82, p = 0.003), suggesting that longer TD correlates with a lower hazard of death (Table 5). Same conclusions arise in the cohort without BRAF or MEK inhibitors (n = 76) with a hazard ratio for TD of 0.81 (p = 0.01) when adjusting for mutation status (Tables 6 and 7).

**Table 5:** Multivariate Analysis of OS in Stage IV in all patients (n = 101) adjusted for treatment duration (TD) and treatment with BRAF/MEK inhibitors.

All patients, n = 101			
Co-variate	Hazard Ratio	95% CI	p-value
Mutation	0.97	0.59–1.62	0.92
TD	0.82	0.71–0.93	0.003
BRAF/MEKi	0.93	0.46–1.88	0.84

**Table 6:** Multivariate Analysis of OSin Stage IV in the subgroup of patients without the BRAF/MEK inhibitors (n = 76).

Without inhibitors, n = 76			
Co-variate	Hazard Ratio	95% CI	p-value
Mutation	1.02	0.61–1.71	0.92
TD	0.80	0.68–0.94	0.009

**Table 7:** Multivariate Analysis of OS from first medication in the subgroup of patients without the BRAF/MEK inhibitors (n = 76).

Without inhibitors, n = 76			
Co-variate	Hazard Ratio	95% CI	p-value
Mutation	0.84	0.51–1.40	0.51
TD	0.85	0.74–0.99	0.04

## Discussion and Conclusion

With the recently introduced targeted therapy for BRAF and NRAS mutated melanomas it led to the question whether the presence or absence of BRAF and NRAS mutation had impact on survival in melanoma patients treated with anti-CTLA-4 antibodies as well.

Our retrospectively analyzed study-cohort, however, did not confirm this hypothesis. In fact,the median OS of 10.12 months in BRAFV600 or NRASmut patients was not significantly longer than the mOSin BRAFV600/NRASwt patients (p = 0.67). These results are in accordance with recently published data by Ascierto et. al [34]. Moreover, previously disease control rate under anti-CTLA-4 Abs has been reported to be mutation independent [31].

Nonetheless, there seems to be a trend for longer OS for NRAS mutated patients as compared to BRAF mutated or BRAFV600/NRASwt patients (p = 0.56). This trend is in accordance with recently published data by Johnson DB et al. elucidating that NRAS mutations can predict for higher objective and clinical responses from immunotherapy compared to BRAFV600/NRASwt melanoma [32]. Although in both retrospective analyses the OS did not reach statistical significance, it seems that NRAS mutated melanoma is more likely to respond to immunotherapy. This is an important observation, as NRAS mutant melanoma is associated with shorter survival [18] and previously showed a correlation with immunotherapeutic agents such as IL-2 [30] and other treatments [35]. Moreover, the introduction of new very promising molecules blocking the immune checkpoints including anti-PD-1 antibodies will need prognostic biomarkers to determine the specific population subset that will benefit from this therapy.

This study also investigated the duration from first distant metastasis to death or till last follow up, termed mOS in stage IV disease. The mOS in stage IV of BRAFV600 or NRASmut patients was also not statistically significant compared to BRAFV600/NRASwt patients ((p = 0.719, HR = 0.91, 95% CI 13–30.3). As a matter of fact there are different publications with discrepant results in this context. For example the publication by Carlino et al. is in accordance with our data and concludes that BRAF and NRAS mutation status do not influence survival in metastatic melanoma [36]. In contrast, Houben et al. found that the presence of either BRAF or NRAS mutations in melanoma patients was associated with poorer survival [9]. All these results including ours have high potential for confounding conclusions with respect to mutation status and overall survival. First of all, there is unquestionably a certain bias in patient selection. In particular, many patients analyzed here were patients included in clinical trials that commonly are fitter, healthier and willing to participate to experimental treatments. Second, the fact that not all BRAFV600 patients in this cohort received targeted therapy, as this new treatment option was not standard of care back in 2006, has probably influenced the findings. Yet, in other large unbiased cohorts of stage I/II and stage IV melanoma patients no prognostic impact could be established [28,29]. Finally, population size was limited in this study and might not have been large enough to see significant trends.

Treatment duration varied across our study population. Only 69% of the patients were able to complete all 4 cycles of anti-CTLA-4 treatment. This is in accordance to previous observations in anti-CTLA-4 therapy, where during administration of immunotherapy high dropout rates, are caused either by adverse reactionsor by rapid disease deterioration [37,38]. Interestingly, the determinant TD had a statistically significant association with OS (HR = 0.82, p = 0.003) and was independent of mutational status. This association suggests that longer TD correlates with a lower risk of death. However, interpretation of this correlation has to be made carefully as undoubtedly slow progressive melanoma is more likely to have longer TD and longer survival as well. Moreover, TD is a factor that cannot be determined a priori and can therefore be determined after administration only. Nevertheless, TD could help to identify the population which is more prone for longer responses and this might affect treatment decisions. Moreover, as it will be difficult for immunotherapy to use progression-free survival (PFS) as surrogate marker for OS [39] it will be interesting to evaluate TD in a prospective setting as a potential surrogate marker for OS in the future.

In concordance to previously reported analysis BRAFV600 or NRAS mutated patients were younger at time of first diagnosis and no difference in sex distribution among the two sub-groups was found [40,41]. One patient had a W616R-BRAF mutation and a G10E mutation in the NRAS oncogene and was classified as BRAFV600/NRASwt. This mutation has to our knowledge never been described before.

In summary, this study has attempted to define a relationship between mutational status and immunotherapy in correlation with survival. It is possible that the rapidly changing treatment algorithms over the past few years have influenced the results of this study causing heterogeneity of the study population. Moreover, sample size and patient selection might have influenced these results. Yet, this study is important as it confirms that mutational status is independent for immunotherapy outcome. In addition, it gives some evidence for better immunologic response in the NRAS mutated population and it supports previously reported characteristics of the BRAFV600 or NRAS population (younger and no difference in gender). Finally, our data suggest that TD could be used as a potential indicator for prolonged survival.
